# The Sensitivity of Fungi Colonising Buckwheat Grains to Cold Plasma Is Species Specific

**DOI:** 10.3390/jof9060609

**Published:** 2023-05-25

**Authors:** Jure Mravlje, Tanja Kobal, Marjana Regvar, Pia Starič, Rok Zaplotnik, Miran Mozetič, Katarina Vogel-Mikuš

**Affiliations:** 1 Biotechnical Faculty, University of Ljubljana, Jamnikarjeva 101, 1000 Ljubljana, Slovenia; tanjakob@gmail.com (T.K.); marjana.regvar@bf.uni-lj.si (M.R.); katarina.vogelmikus@bf.uni-lj.si (K.V.-M.); 2 Jozef Stefan Institute, Jamova 39, 1000 Ljubljana, Slovenia; pia.staric@ijs.si (P.S.); rok.zaplotnik@ijs.si (R.Z.); miran.mozetic@ijs.si (M.M.)

**Keywords:** non-thermal plasma, decontamination, disinfection, crops, *Alternaria*, *Aspergillus*, *Cladosporium*, *Epicoccum*, *Fusarium*

## Abstract

Fungi are the leading cause of plant diseases worldwide and are responsible for enormous agricultural and industrial losses on a global scale. Cold plasma (CP) is a potential tool for eliminating or inactivating fungal contaminants from biological material such as seeds and grains. This study used a low-pressure radiofrequency CP system with oxygen as the feed gas to test the decontamination efficacy of different genera and species commonly colonising buckwheat grains. Two widely accepted methods for evaluating fungal decontamination after CP treatment of seeds were compared: direct cultivation technique or contamination rate method (%) and indirect cultivation or colony-forming units (CFU) method. For most of the tested fungal taxa, an efficient decrease in contamination levels with increasing CP treatment time was observed. *Fusarium graminearum* was the most susceptible to CP treatment, while *Fusarium fujikuroi* seems to be the most resistant. The observed doses of oxygen atoms needed for 1-log reduction range from 10^24^–10^25^ m^−2^. Although there was some minor discrepancy between the results obtained from both tested methods (especially in the case of *Fusarium* spp.), the trends were similar. The results indicate that the main factors affecting decontamination efficiency are spore shape, size, and colouration.

## 1. Introduction

We are facing an unprecedented increase in the number and spread of disease-causing fungi due to human activity such as globalisation, causing long-distance dispersal of fungal propagules, and also due to environmental and climate changes [1]. Fungal diseases present one of the main concerns and potential threats at all stages of cereal crop production, from preharvest to postharvest processes and storage [2]. They can suppress germination and cause spoilage of stored grains [3] by discolouration, reducing their baking and cooking quality, lowering nutritional value, causing unwanted odors, rotting, and developing allergenic propagules [4,5]. Some fungi can also produce toxic secondary metabolites known as mycotoxins that are harmful to humans and animals [6], with a profound negative effect on whole plant production and food security. Fungi can colonise grains whenever the temperature and humidity conditions are favourable [7], and once onset, it is very challenging to remove them as their cells and spores are very resistant [8]. Filamentous fungi are ubiquitous and can colonise grains in the field (preharvest or field fungi) or during storage (postharvest or storage fungi) [9]. The fungal genera *Alternaria, Aspergillus, Cladosporium, Epiccoccum*, and *Fusarium* are common colonisers of buckwheat grains in the natural environment [10,11,12,13,14]. They can live as endophytes inside plants or as pathogens, causing plant diseases. Especially problematic are those that can synthesise mycotoxins, such as aflatoxins and ochratoxins (*Aspergillus* sp.), fumonisins, trichothecenes and zearalenone (*Fusarium* sp.) and *Alternaria* toxins [15,16,17].

Worldwide, fungal infections threaten our food security by reducing the yields of crop plants or even causing a complete decay of plants [18]. With a rapidly growing population estimated to reach ten billion in 2050 [19], it is essential to prevent fungal growth to achieve both food safety and security on a global scale. Postharvest applications of chemical fungicides by spraying or wax-coating are still the most commonly used commercial practices [8,20]. However, these methods leave residues and can negatively impact our environment and health and are therefore becoming less popular and acceptable among consumers [21]. Moreover, the increased fungal resistance to all licensed systemic antifungal agents, including fungicides [22], is driving a growing demand and interest in seeking new, natural and environmentally friendly antifungal agents or safe and sustainable technologies for preventing fungal growth.

In the last few decades, the interdisciplinary research field of cold or non-thermal plasma technology has emerged with rapidly developing applications in many scientific areas, including agriculture, biotechnology, and the food industry. A new, rapidly developing multidisciplinary field of research called» plasma agriculture« has been established [23,24]. Surface decontamination of grains before sowing or using them as food is the most promising among the applications of cold plasma technology. Several comprehensive review papers have been published recently, summarising state-of-the-art fungal decontamination of cereal grains [25,26,27] and mycotoxin degradation by cold plasma technology [28,29].

Cold plasma (CP) refers to the fourth state of matter, a partially ionised gas generated by applying thermal or electrical energy to a gas [30]. It is a »quasi-neutral« medium (with a zero net charge) but conducts electricity as it contains free-charged particles that give its unique properties [31]. It consists of free electrons, molecules, atoms, ions, radicals, and other reactive species and represents a source of radiation, in particular, ultraviolet (UV) and vacuum ultraviolet (VUV) photons [32]. All these chemically active species have a profound antimicrobial effect and can be used for surface bio-decontamination [33,34].

Our previous studies used low-pressure CP technology to decontaminate buckwheat grains. After CP treatment, we showed significant changes in the frequency and diversity of naturally occurring fungal colonisers of buckwheat grains [14]. Furthermore, we also demonstrated that direct exposure to CP (in »glow« mode) is more effective in the decontamination of grains than indirect exposure (»afterglow« mode) [35]. However, in the research of naturally occurring fungi on grains (natural grain microbiota), the absence of fungal taxa can be attributed to the successful CP decontamination or the absence of fungi. Therefore, we designed an experiment using a uniform single fungal species inoculation method to discriminate between these possibilities. The procedure involved fungal inoculation of sterilised buckwheat grains using uniform spore concentrations (10^6^ spores mL^−1^). The main aim of this research was to evaluate the effects of low-pressure CP on specific fungal species or taxons that are common colonisers of buckwheat grains in the natural environment, such as *Alternaria*, *Aspergillus*, *Cladosporium*, *Epiccoccum*, and *Fusarium* species. This study intends to contribute to a better understanding of the effect of the low-pressure CP experimental setup device on specific fungal taxa, presenting a key point for further unravelling the CP decontamination mechanisms at a single species level.

## 2. Materials and Methods

### 2.1. Source Material

**Figure 1:**
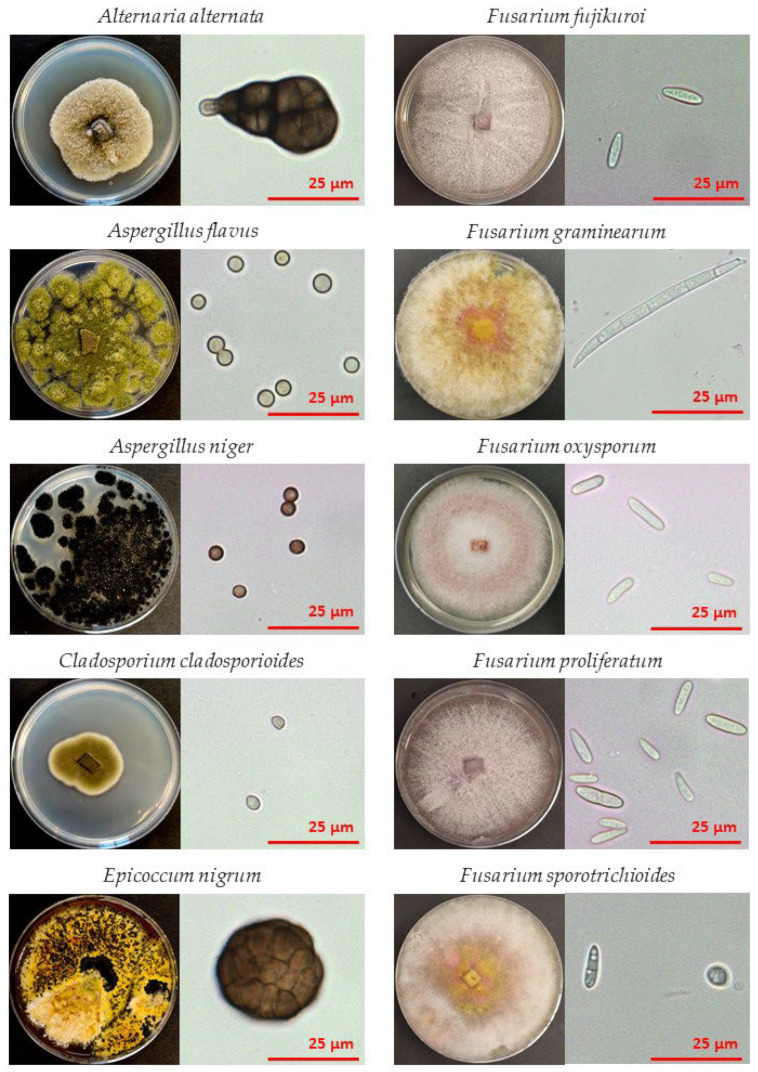
Fungal species used in our study. A photograph of each 1-week-old fungal culture on PDA medium after incubation at 24 °C in the dark (left column). Each fungal culture’s accompanying spores (micro- or macroconidia; optical microscope photographies) after 7–10 days of growth, depending on species: (right column).

Grains of common buckwheat (*Fagopyrum esculentum* Moench) were obtained from Rangus Mill (located in Šentjernej, south-eastern Slovenia, about 230 m a.s.l.). They were harvested in 2021 and stored in a dry and dark environment at room temperature until the experiments were performed in 2022. Fungal strains used in these experiments were previously isolated from buckwheat grains in our laboratory, grown in pure cultures, and morphologically and/or molecularly characterised [11,14,35], deposited and stored in the fungal bank of the Laboratory for Plant Physiology at the Chair of Botany and Plant Physiology, Department of Biology, Biotechnical faculty, University of Ljubljana. The strains used were: *Alternaria alternata* (GB002), *Aspergillus flavus* (GB005), *Aspergillus niger* (GB006), *Cladosporium cladosporioides* (GB007), *Epicoccum nigrum* (GB009), *Fusarium fujikuroi* (GB011), *Fusarium graminearum* (GB012), *Fusarium oxysporum* (GB013), *Fusarium proliferatum* (GB014), and *Fusarium sporotrichioides* (GB015) (Figure 1).

### 2.2. Uniform Single Fungal Species Inoculation Method for Artificial Contamination of Buckwheat Grains

To ensure equal loads of each fungal species for the inoculation, fungi were inoculated on 2% potato dextrose agar (PDA) supplemented with the antibiotic chloramphenicol (50 mg L^−1^) to prevent bacterial contamination and incubated in growth chambers at a constant temperature of 24 °C in the dark until sporulation (7–10 days—depending on species). A spore suspension of each fungus was prepared by flooding 5 mL of sterilised saline solution (0.9% NaCl) with 0.1% (*v*/*v*) aqueous Tween 80 and gently scraped using a sterile glass stick. The spores were counted under an optical microscope (Carl Zeiss) with a cell-counting haemocytometer (Neubauer chamber: Assistant Bright Line). Before application, the spore concentration was adjusted to 10^6^ spores mL^−1^.

Buckwheat grains were sterilised by autoclaving (121 °C, 15 min) in Systec VX-150 autoclave, and samples of 10 g were inoculated with 10 mL of fungal spore suspension in a 150 mL flask and vigorously shaken on a rotatory shaker (180 rpm) for 30 min to distribute fungal spores uniformly. After the inoculation, the grains were transferred to sterile Petri dishes, spread evenly, and let air dry for 24 h under a laminar flow hood.

### 2.3. Cold Plasma Treatment Conditions

Inoculated buckwheat grains were treated with a low-pressure CP in a large reactor described in detail earlier [36]. In short, it is a 2 m long reactor powered by inductively coupled discharge. Plasma is generated inside an excitation coil connected to a 27.12 MHz radiofrequency (RF) generator (Induktio UHFG-8) through an L-type impedance-matching network built for the purpose. The excitation coil was wrapped around a glass vacuum discharge chamber 20 cm in diameter and 2 m in length. The vacuum chamber was pumped with a two-stage rotary pump Leybold Trivac D65B, with a nominal pumping speed of 65 m^3^ h^−1^. The oxygen of 99.999% purity was leaked into the vacuum chamber on the opposite side of the pumping duct using the Aera FC7700 Advanced Energy mass flow controller. The pressure was measured with an absolute capacitance manometer MKS Baratron type 722B.

Buckwheat grains (about 5 g per batch) were spread on a perforated aluminium holder and placed into the pumped vacuum chamber. Relatively high pressure persisted due to the desorption of water vapour from the grains. After the pressure of 15 Pa was reached, oxygen was leaked into the chamber at the rate of 50 sccm so that the pressure of 30 Pa was maintained. The 1.8 kW plasma generator was turned on, and grains were exposed to plasma for 30, 60, 120 or 180 s. At the selected discharge parameters, the density of oxygen atoms was around 2.5 × 10^21^ m^−3^ at the position where grains were treated. Neutral oxygen density was measured with a cobalt catalytic probe. A detailed description of catalytic probes can be found in Zaplotnik et al. [37].

**Figure 2:**
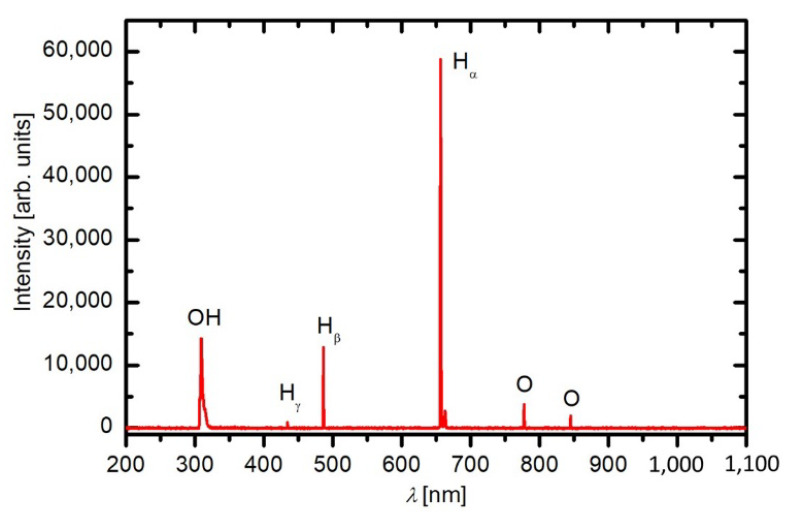
Typical optical emission spectrum of a low-pressure plasma during the buckwheat grain treatment. The integration time is 100 ms.

Figure 2 shows a typical optical spectrum of the plasma during the treatment of the grains. Optical spectra were acquired with Avantes AvaSpec 3648 fiber optic spectrometer with a 0.3 nm resolution over the range from about 200 to 1100 nm. The most intense spectral features arise from atomic transitions, which indicate a high dissociation of gaseous molecules in the discharge chamber. Apart from oxygen atom lines at 777 nm and 845 nm, hydrogen lines at 656, 486 and 434 nm and OH emission systems at around 309 nm can be observed. The hydrogen and OH spectral features confirm the presence of water vapour inside the discharge chamber, dissociated in plasma. Although the oxygen was continuously leaked into the discharge chamber, plasma was also rich in other chemically reactive species. Therefore, using the expression “oxygen plasma” for the experimental system loaded with many grains is inappropriate. Plasma in our device was thus generated in a mixture of oxygen and water vapour.

### 2.4. Evaluation of the Efficiency of the Cold Plasma Decontamination

#### 2.4.1. Direct Cultivation Technique: Contamination Rate

For evaluating the decontamination efficiency using the direct evaluation method (contamination rate), the agar plate or “Ulster” method was used [38]. Buckwheat grains were plated on sterile plastic Petri dishes (diameter 90 mm) filled with 2% potato dextrose agar (PDA) supplemented with the antibiotic chloramphenicol (50 mg L^−1^) to prevent bacterial growth. Ten buckwheat grains from each treatment in the group, including the control and all treatment times for each fungus, were evenly distributed to each Petri dish to assess fungal growth. For each of the treatments in the group, ten replicates were prepared. Plates were incubated in growth chambers at 24 °C in the dark for one week. After that time, the contamination rate (CR) was calculated as follows:

CR (%) = (number of colonised grains)/(total number of grains) × 100
(1)

#### 2.4.2. Indirect Cultivation Technique: Colony-Forming Units (CFU)

The indirect evaluation method is based on the colony-forming units (CFU) count used to evaluate decontamination efficacy. First, 1 g of buckwheat grains from each treatment in the group were placed in 150 mL sterile flasks, and 9 mL of sterilised saline solution (0.9% NaCl) with 0.1% (*v*/*v*) aqueous Tween 80 was added. Next, the flasks were shaken for 30 min on a rotatory shaker (180 rpm). After that, the washings were serially diluted with saline solution to the appropriate concentration (ten-, hundred- or thousand-fold depending on each fungus) and 100 µL of diluted solutions were plated on agar plates (diameter of 90 mm, filled with 2% PDA supplemented with the antibiotic chloramphenicol (50 mg L^−1^) to prevent bacterial growth). After five days of cultivation in growth chambers at a temperature of 24 °C in the dark, the colony-forming units were counted and expressed as CFU count g^−1^ of grain.

### 2.5. Germination Tests

To estimate the effect of each CP treatment on the germination of buckwheat grains, non-sterilised (not autoclaved) grains were used. In germination tests, 20 grains from each CP treatment were placed into Petri dishes (diameter 70 mm) on two layers of filter paper and moistened with 3 mL of sterile distilled water. For each group of grains, the germination test was performed in five replicates. The grains were incubated in plant growth chambers at 24 °C, with 60% humidity, in the dark. The number of germinated grains was counted after one week of incubation. A visible penetration of the radicle through the seed coat was used as the criterion for germination. The germination rate (GR%) was calculated as follows:

GR (%) = (number of germinated grains)/(total number of grains) × 100(2)

### 2.6. Statistical Analysis

All results are expressed as mean ± standard error (SE) of a set number of replicates: 10 for contamination rate, 5 for germination tests and 3 for CFU counts. Statistical significance between groups of fungi with different treatments was determined using one-way analysis of variance (ANOVA) with Duncan’s post hoc test (using Statistica StatSoft version 7). The significance level was considered at a *p*-value of less than 0.05.

## 3. Results

### 3.1. Cold Plasma Decontamination Efficiency

#### 3.1.1. Direct Cultivation Technique: Contamination Rate

**Figure 3:**
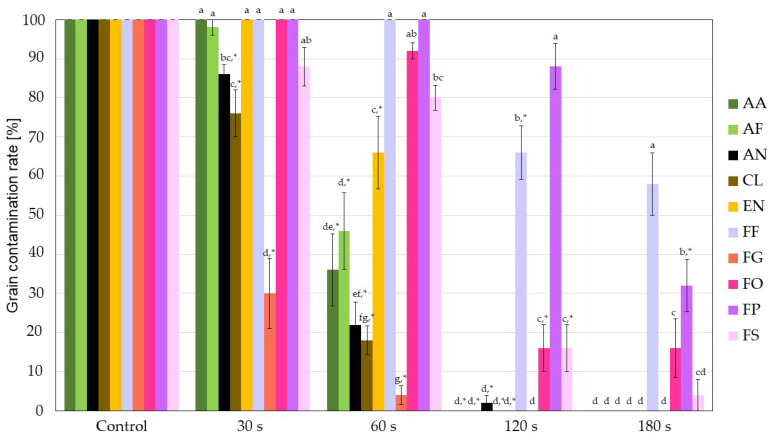
Grain contamination rate (%) for each fungal strain. Different letters (superscripts) indicate statistically significant differences between different groups of fungi per each treatment. (*) indicate a statistically significant decrease from the respective previous treatment. AA—*Alternaria alternata*; AF—*Aspergillus flavus*; AN—*Aspergillus niger*; CL—*Cladosporium cladosporioides*; EN—*Epicoccum nigrum*; FF—*Fusarium fujikuroi*; FG—*Fusarium graminearum*; FO—*Fusarium oxysporum*; FP—*Fusarium proliferatum*; FS—*Fusarium sporotrichioides*.

The uniform single fungal species inoculation method proved very successful, as in control groups, a 100% grain contamination with selected fungal species was achieved with all tested fungi (Figure 3). After 30 s cold plasma treatment (CPT), significant differences in sensitivity to CPT compared to respective controls (marked with * in Figure 3) were observed in *Aspergillus niger*, *Cladosporium cladosporioides* and *Fusarium graminearum*, with the latter being the most efficiently inactivated. Even more pronounced were the differences between fungi after 60 s CPT, where more than a 50% reduction in contamination rate was observed for half of the tested fungi, namely *Alternaria alternata*, *Aspergillus flavus*, *A. niger*, *C. cladosporioides* and *F. graminearum*. Again, *F. graminearum* was the most strongly affected by CPT, as less than 5% of grains remained contaminated. An approximately 35% decrease in contamination rate was also observed for *Epicoccum nigrum*. In the listed fungi, significantly less grain contamination was observed after 60 s CPT than at 30 s CPT. In the case of other *Fusarium* spp., 60 s CPT was the least efficient, resulting in only up to 20% decontamination rate. Among these, the 60 s CPT had the most influence on *F. sporotrichioides*, being the only one that differed significantly from its control.

After the 120 s CPT, *A. alternata*, *A. flavus*, *C. cladosporioides*, *E. nigrum* and *F. graminearum* were completely inactivated, and only a few grains remained contaminated with *A. niger*. Again, the other four species from the genus *Fusarium* showed more tolerance to CPT, with *F. oxysporum and F. sporotrichioides* being more successfully disinfected, as only around 15% of grains remained contaminated. In the case of *F. fujikuroi*, a 35% decontamination rate was observed. The 120 s CPT proved the least efficient towards *F. proliferatum*, as only a little more than 10% decontamination was observed. It was the only fungus that did not statistically differ from its control even after 120 s CPT.

**Figure 4:**
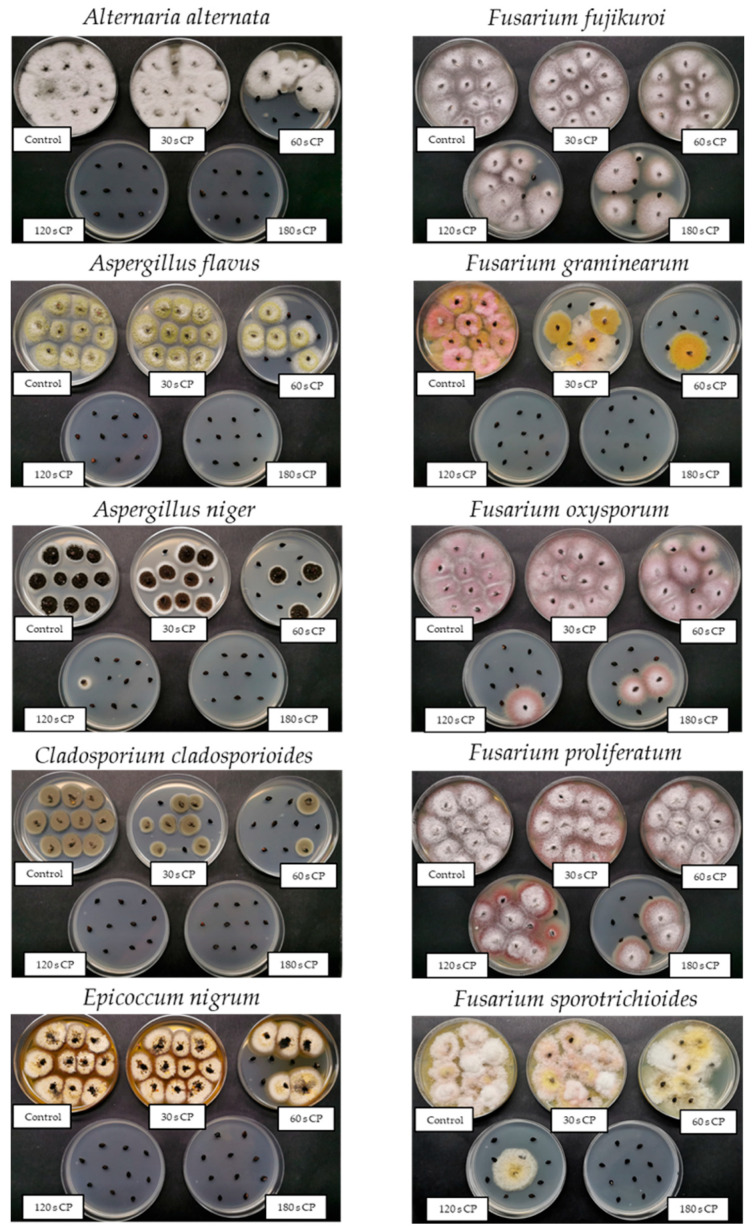
Representative photographs of the treatments: control grains infected with selected fungus and each cold plasma (CP) treated group of grains pre-infected with selected fungus.

Interestingly, after 180 s CPT, we found a better decontamination rate in the case of *F. proliferatum* (around 70% compared to its control) than in *F. fujikuroi*, which was still less than 50%. *F. proliferatum* was also the only fungus from the genus *Fusarium* that was statistically more effectively decontaminated after 180 s CPT compared to 120 s CPT (for more than 50%). Representative photographs of each control and each CPT group are shown in Figure 4.

#### 3.1.2. Indirect Evaluation Method: Colony Forming Units (CFU)

In Table 1, absolute numbers of colony-forming units (CFU) for control and each CPT for each fungus are expressed as a logarithm of CFU per gram of grains. Note that all the grains were inoculated with 10^6^ conidia of each fungus as described in the section Materials and Methods. It should be stressed that although grains were inoculated with the same number of conidia from different fungi, in the same way, their attachment rates were quite different (in the range of 3.5 to 6.3-log CFU g^−1^ grains).

After 30 s CPT, significantly lower counts than for controls were observed in the case of *A. flavus, A. niger, F. fujikuroi, F. oxysporum* and *F. sporotrichioides.* Interestingly, for *F. graminearum,* no fungal growth was observed, although the direct evaluation (Figure 4) showed a measurable degree of seed contamination.

After 60 s CPT, differences were observed in all fungi compared to their controls, and, except for *A. niger*, all fungi were also more efficiently decontaminated than with 30 s CPT. After 120 s CPT, all other fungi than the remaining *Fusarium* spp. were decontaminated entirely from the surface of the grains as no CFUs were found. The CFU reduction ranged from almost 3-log (in *F. fujikuroi*) to less than 2-log (in *F. oxysporum*) units. Interestingly, after 180 s CPT, no further decontamination was observed in *Fusarium* spp., except for *F. oxysporum*.

**Figure 5:**
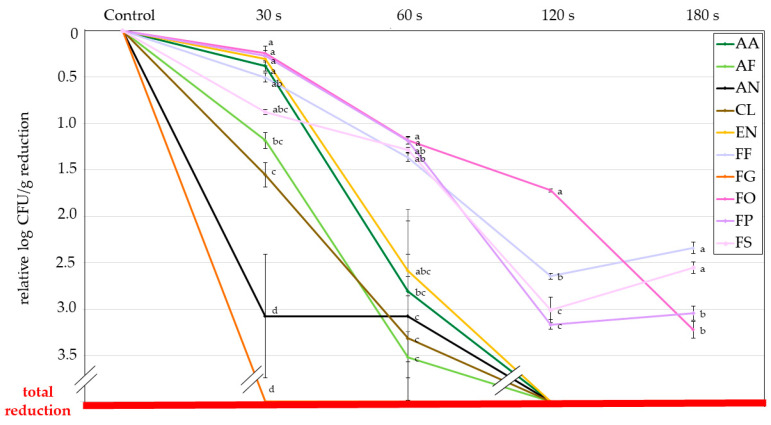
Indirect evaluation of fungal growth expressed as relative log CFU g^−1^ reduction per treatment for each fungus. The red line indicates the total reduction of fungal growth. Different letters (superscripts) indicate statistically significant differences between different groups of fungi per each treatment. AA—*Alternaria alternata*; AF—*Aspergillus flavus*; AN—*Aspergillus niger*; CL—*Cladosporium cladosporioides*; EN—*Epicoccum nigrum*; FF—*Fusarium fujikuroi*; FG—*Fusarium graminearum*; FO—*Fusarium oxysporum*; FP—*Fusarium proliferatum*; FS—*Fusarium sporotrichioides*.

Due to the different attachment efficacies described above, all results presented below in this document are expressed relative to each fungus control group; therefore, the results are comparable between species. Figure 5 shows the contamination control efficiency of cold plasma expressed as the relative logarithmic CFU per gram of grains reduction for each fungus. The highest sensitivity to CPT was observed in the case of *A. niger* and *F. graminearum*, reaching more than 3-log CFU g^−1^ reduction after 30 s CPT. *F. graminearum* was completely inactivated or removed. In other species, differences were less pronounced, and their reduction levels were from almost 0 to 1.5-log CFU g^−1^. Interestingly after 60 s CPT, most tested fungi could be grouped into two groups, with other *Fusarium* spp. Being more tolerant to CPT (their reduction levels were only around 1-log unit), while both *Aspergillus* species and *C. cladosporioides* showed much higher sensitivity, on average more than 3-log units. Results obtained for *A. alternata* and *E. nigrum* were somewhere in between.

According to the direct evaluation method, a similar trend was observed after 120 s CPT, as all fungal species except the remaining *Fusarium* spp. were decontaminated entirely. However, contrary to the direct evaluation method, in the CFU method, CPT showed the least efficiency in removing *F. oxysporum* (only 1.7-log CFU g^−1^ reduction compared to the control), followed by *F. fujikuroi* (2.6-log CFU g^−1^ reduction). In this case, *F. proliferatum* and *F. sporotrichioides* would be considered the least resistant to CPT (both reduced for around 3-log CFU g^−1^ compared to the control). In contrast, after 180 s CPT, *F. oxysporum* was eradicated way more efficiently than the other three *Fusarium* spp. compared to 120 s of CPT. After 180 s of CPT *F. fujikuroi* and *F. proliferatum* were found to be the most resistant to CPT, their reduction rate was only around 2.5-log CFU g^−1^ compared to the control.

**Figure 6:**
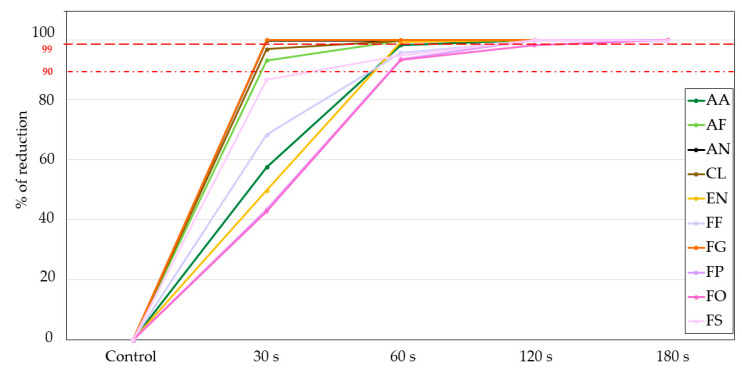
Fungal growth rate after cold plasma treatment expressed as a percentage of reduction per treatment for each fungus. The first dotted red line indicates a 90% reduction in fungal growth (1 log reduction), and the second dotted line indicates a 99% reduction in fungal growth (2 log reduction). AA—*Alternaria alternata*; AF—*Aspergillus flavus*; AN—*Aspergillus niger*; CL—*Cladosporium cladosporioides*; EN—*Epicoccum nigrum*; FF—*Fusarium fujikuroi*; FG—*Fusarium graminearum*; FO—*Fusarium oxysporum*; FP—*Fusarium proliferatum*; FS—*Fusarium sporotrichioides*.

Another measure for estimating the efficacy of CPT for reducing microbial growth is presented in Figure 6. Here we show the percentage of the reduction of microorganisms (fungi), which is also a typical microbiological measure for providing decontamination efficacy after sterilising treatment. The first dotted red line corresponds with a 90% reduction rate (1 log), and the second dotted red line with a 99% reduction rate (2 log). From there, we see that for around half of the tested fungi, we achieved a 99% reduction rate already with 60 s CPT treatment (for *A. niger* and *F. graminearum* already with 30 s CPT). For *A. alternata* and all *Fusarium* species, except for *F. oxysporum*, a 99% reduction was achieved after 120 s CPT. After 180 s CPT, a 99,9% reduction (3 logs) was demonstrated for *F. oyxsporum* and *F. proliferatum*, while the reduction rate of the growth of *F. fujikuroi* and *F. sporotrichioides* remained at 99% (2 logs).

### 3.2. Effect of Cold Plasma on Grain Germination

The effect of our CPT on the germination ability of buckwheat grains was also evaluated. Germination tests were performed on non-autoclaved grains. The results (Table 2) show that the germination rate decreased with increasing CPT time. After 30 s CPT, 72% of grains still germinated, while after 60 s CPT, only around half of the grains were able to germinate. Already 120 s of CPT almost entirely ceased the germination, and no grains germinated after the longest (180 s) CPT.

## 4. Discussion

In the last two decades, the research on various fields of fungal decontamination by CP has been thoroughly examined in the most recently published review papers [25,39]. However, these experiments were performed on various objects, each practically treated in different CP setup devices under various parameters. Besides, only a few selected fungi were usually tested, especially when dealing with artificially infected material. We still lack a comprehensive understanding and establishment of a database of biological responses of a broader spectrum of fungal species under various CP setups, parameters, and intensities. Bearing that in mind, our study aimed to examine the response of different fungal genera and species from the same genera that colonise grains of common buckwheat to our cold plasma setup device. Moreover, we also wanted to test if different evaluation methods commonly reported in CP decontamination studies, i.e., direct and indirect cultivation techniques, can affect the outcome of the reported results.

It is agreed that the mechanisms of action in CP decontamination are entirely different from those of classical sterilisation techniques. The antimicrobial effect is achieved either by UV radiation or by etching via ions and chemically reactive plasma species reacting with the surface of the treated material or microorganism [33]. All plasma species cause cell wall damage and oxidation of cellular membranes and proteins, leading to the disintegration and leakage of cells [40]. Once the outer layers are disrupted, and cellular integrity is compromised, the UV photons can also directly damage genetic material [41]. However, their penetration depth is minimal due to the cover of numerous cellular debris. It is believed that in atmospheric-pressure air plasmas, reactive species play the most crucial role in microbial decontamination [42]. However, in low-pressure plasma systems, there is an essential contribution of UV photons in the sterilisation process [33,43,44]. In addition, a thermal effect due to sample heating can also occur in a low-pressure plasma reactor [45]. So the mechanisms involved and their contributions to the microbial inactivation are various and primarily dependent on the parameters of the CP, but also the environmental factors and the species and properties of the treated microorganisms play a crucial role in the outcome [46]. Especially when dealing with filamentous fungi, it can be hard to compare the effects of CPT between different species as they are very diverse in their hyphae morphology, spore structure and biochemical processes.

Firstly, we must point out that the attachment efficacy among fungal spores differs. This can be due to the differences in size and shape of spores (conidia) of fungal species, which most likely affects their interaction with the surface of the buckwheat grains. So, all results related to the CFU method in our article are expressed relative to the control CFU count of each fungus, so they are comparable between species. In our case, *Fusarium graminearum* was found to be the most susceptible to CPT, regardless of the method used for evaluation. Still, we observed quite a significant discrepancy when comparing both methods (Figure 3 and Figure 6). The CFU method observed the complete reduction after 30 s CPT.

In comparison, there was still some minimal (less than 5%) growth even after 60 s of CPT when estimating via the contamination rate method. This could be partially attributed to a lower attachment efficacy; however, there is quite a big difference if we compare the results with *Alternaria alternata,* which showed almost the same attachment rate in the control. So, CPT must have a greater impact on *F. graminearum* than on *A. alternata*. Similarly, differences in the initial spore inoculum were already pointed out as a possible factor for CP decontamination efficacy; however, other factors, e.g., cellular and molecular differences such as pigments, can also be crucial [47]. Some studies already showed that *F. graminearum* is quite easily eradicated with atmospheric-pressure CPT [47,48], the afterglow of the low-pressure CP [49] and plasma-activated water [50]. That is a good sign, as *F. graminearum* is known to be one of the primary pathogens and mycotoxin-producing fungi infecting cereals in Europe and worldwide [51].

In the “second group” of fungi, based on their susceptibility to CP, when combining the results of both evaluation methods, both *Aspergillus* spp. and *Cladosporium cladosporioides* could be classified. Fungi from the genus *Aspergillus* are, besides *Penicillium* spp., one of the main mycotoxin-producing genera that contaminate food and feed during storage [52]. It is thus not surprising that they are amongst the most studied genera in CP decontamination research. Numerous studies report a successful removal of *A. flavus* [53,54,55,56,57,58,59,60] and *A. niger* [61,62,63,64,65] with CP either in suspension or on seeds and fruits. In our study, both contamination levels of grains with *Aspergillus* spp. were significantly decreased after 30 s CPT (based on the CFU method), and more than 50% decontamination efficacy was achieved after 60 s CPT. Furthermore, both species were completely eradicated after 120 s CPT (based on the results of the CFU method), and less than 5% of grains remained contaminated with *A. niger* when testing the grain contamination rate.

On the other hand, there is less evidence of CP decontamination of *Cladosporium cladosporioides* up to this point. One study reported complete inactivation of *C. cladosporioides* spores in suspension after 1 s CPT with a powerful microwave plasma jet [64]. A significant reduction of *C. cladosporioides* by 1-log CFU g^−1^ was also reported when treating dried filefish fillets with oxygen CP [66]. However, there were some other reports about relatively good inhibition of different *Cladosporium* spp. in suspension [67] and on seeds various using atmospheric-pressure CP devices [68,69]. Our results show that *C. cladosporioides* was significantly reduced after 30 s CPT (based on the results of both methods) and even more efficiently after 60 s CPT, as less than 20% of grains remained contaminated. Similarly, for both *Aspergillus* spp., complete decontamination was achieved after 120 s CPT.

*Alternaria alternata* was found to be somewhat more resistant to CPT in the initial 30 s treatment when compared to *Aspergillus* spp. *and C. cladosporioides*, which is especially evident in the results of the CFU method. That could be attributed to their specific conidia structure, which may be multicellular (on average containing 3–7 cells per conidium). It was already suggested that a higher cell number in the spore structure could provide an advantage during CP treatment, as there are more chances for cells to escape from being sterilised [42,47]. This is because multicellular spores (conidia) may still have the possibility to germinate if not all the cells in the spore (conidium) are killed by CPT [42]. After 60 s CPT, we observed no significant differences between the above fungal taxa, which could be because the conidial structure of *A. alternata* has probably already been destroyed after 30 s CPT and those spores that were able to survive the 30 s CPT were more easily eradicated with 60 s CPT. Similar results were obtained by Zahoranova et al. 2018 when testing the efficacy of atmospheric CPT on artificially contaminated maize seeds. They found *Fusarium culmorum,* which is phylogenetically very close to the *F. graminearum* species complex [70] and has very similar macroconidia with six cells, on average, to be the most sensitive to CPT followed by *A. flavus* and *A. alternata*. Their results also show no CFU of *A. alternata* observed after 300 s CPT; however, some maize seeds were still colonised with *A. alternata* when estimating by the contamination rate method (and the same in the case of *F. culmorum* after 60 s CPT), which is like our findings regarding *F. graminearum*. They also pointed out that seed surface topology and cracks can shelter fungal spores, protecting them against CPT and the washing procedure needed for the CFU method, making it impossible to determine viable fungal spores by the CFU method. However, even one viable fungal spore on the seed can manifest in a fungal infection, which can be detected with a direct cultivation method. All these results indicate that the CFU method may not be the most reliable to judge the complete decontamination of seeds and that accompanying contamination rate tests should be performed to confirm the findings.

Similar behaviour as in *Alternaria alternata* was observed for *Epicoccum nigrum*. No decontamination effect was observed after 30 s CPT; after 60 s CPT, more than 2.5-log CFU g^−1^ reduction was observed. Again, the contamination rate method showed less efficient decontamination as fungal growth was not observed in only around 35% of grains. This greater tolerance to CPT could be attributed to more cells per conidium, like *A. alternata*, but also to the fact that the spores are very large and thick, even more than those of *A. alternata* (Figure 1). The thickness could also be the reason why *E. nigrum* proved more tolerant to CP treatment, at least according to the contamination rate method, as the thickness of the microorganisms to be inactivated is also known to affect the efficacy of sterilisation strongly [44]. To our knowledge, no studies have been performed on the CP treatment of *E. nigrum*. But since *E. nigrum* is a ubiquitous saprophytic fungus and a typical plant endophyte colonising various plants in nature [71], we believe it should be further investigated.

All these fungi (*Alternaria, Aspergillus, Cladosporium* and *Epicoccum*) also possess one common characteristic—they have highly pigmented conidia (spores) with melanin [72,73,74], which is evident in Figure 1. Melanins are pigments that have a well-known role in the pathogenesis of microorganisms. They also confer tolerance to extreme environments (high insolation, extreme temperatures and low water activity) and damaging agents such as UV, ionising, gamma irradiation, toxic metals, hydrolytic enzymes, antifungal drugs and free oxygen radicals [75,76]. Furthermore, by their photochemical properties, they may interact with a wide range of electromagnetic radiation frequencies, functioning as a protective and energy-harvesting pigment [75]. Similarly, dark-coloured (melanised) conidia of *Aspergillus carbonarius* and *Alternaria alternata* have shown a higher resistance to the atmospheric-pressure CPT than lighter-pigmented and thinner cells of *Botrytis cinerea* and *Monilinia fructicola* [42]. Therefore, the authors assumed that UV photons generated at the atmospheric-pressure CP did not significantly contribute to the direct inactivation of fungi. Instead, they concluded that the difference in the efficacy of surface etching was more likely related to the cell wall structure and thickness affecting mechanical and chemical strength, as well as to the stacking of spores on their surfaces and the number of cells per spore. But since fungal melanins’ roles include scavenging free radicals and giving mechanical and chemical cellular strength [75], they could have a profound role in fungal tolerance to CP.

*Fusarium oxysporum* is a common soil fungus that can be a beneficial endophyte or pathogen for many predominantly agriculturally essential plants. Artificially inoculated scots pine seeds with *F. oxysporum* have been treated with plasma sustained by a diffuse coplanar surface barrier discharge (DCSBD). Almost 100% decontamination efficacy was achieved after 30–60 s [77]. However, the germination of pine seeds was also dramatically reduced, as only around 5% of seeds still germinated after 30 s CPT. The authors recommended the optimum treatment of 3 s: after such a short treatment, most seeds remained alive, and the decontamination efficacy was more than 90%. Less than 10% survival of the spores was also observed for *F. oxysporum* f. sp. *lycopersici* after being treated with dielectric barrier discharge (DBD) in saline for 10 min [78]. It has been demonstrated that treatment with atmospheric pressure CP jet using helium as a feeding gas did not significantly reduce the mean mycelial growth rate or virulence of the *F. oxysporum* f. sp. *basilici*. In contrast, CP jet treatments on seedlings and a CP DBD treatment of seeds exhibited varying efficacy against *F. oxysporum* f. sp. *basilici* [79]. 

On the other hand, it has been shown that *F. oxysporum* hyphal growth was successfully inhibited with a microwave plasma jet when oxygen was used in plasma generation [47]. Furthermore, complete inhibition of *F. oxysporum* mycelial growth and spore germination was also achieved after a 90 s treatment using an air plasma jet [80]. This research also showed that the efficacy of decontamination of the spores of the same species on the surface of pepper was only around 50% at the same CPT conditions. This indicates that fungi are more easily eradicated in suspension or when put on a flat surface and treated with CP rather than on some biological material such as seeds. It was already pointed out that the surface structure of the treated material can lead to non-homogenous sterilisation [81]. Previous reports also indicated that the decontamination efficacy depends on the type of contaminated seeds, with shape and surface playing a significant role [4,82], as seed wrinkles and crevices have already been mentioned as a possible factor limiting the efficiency of CPT decontamination [14,83,84]. This phenomenon was also demonstrated for mycotoxin degradation by CPT, and it was concluded that the reactive species produced in plasma could be scavenged by the different components of the substrate leading to less efficient decontamination [85]. Also, when dealing with mycotoxin-contaminated cereals and nuts, besides plasma source and treatment time, a significant role was contributed to the type (especially surface properties) of the treated cereals and nuts [57,59,86,87]. In accordance with our results, conidia of *F. oxysporum* were shown by other authors to be significantly more tolerant to CPT than *F. graminearum*, suggesting that microconidia could be more tolerant to CP stress than macroconidia [47].

Similarly, as in the case of *F. graminearum*, the results of the CFU evaluation method showed better decontamination efficacy, as 30 and 60 s CPT already caused a statistically significant reduction in germination. At the same time, the difference in the contamination rate became substantial only after 120 s CPT. This could be attributed to the high initial load of fungal spores (as the inoculation of buckwheat grains with 10^6^ conidia governed almost 6-log CFU g^−1^ in the control group), indicating a very good attachment rate of this fungi. However, it could be concluded that even if more than 1-log CFU g^−1^ reduction was achieved, as in the case of 60 s CPT, that did not mean a significant biological relevance, as there was still more than 90% contamination rate observed in buckwheat grains. Similarly, when comparing the results of 120 s CPT and 180s CPT, we observed 1.5-log CFU g^−1^ reduction, but again no real difference in achieved decontamination rate efficacy.

To our knowledge, there have been no tests on the efficacy of the CP decontamination of *Fusarium sporotrichioides*. But CPT should target it since it is one of the most important fungal pathogens in temperate and tropical regions, especially in wheat), capable of producing various mycotoxins [88]. CPT should target it. Interestingly, *F. sporotrichioides* was the only fungal species where the results of the contamination rate method showed better decontamination efficacy than the CFU method, contrary to *F. graminearum*. Besides the fact that the conidia of *F. sporotrichioides* had better initial attachment efficacy than the conidia of *F. graminearum* for more than 1-log CFU g^−1^, this could also be due to their smaller size and thus possibly better detectable in the CFU counting method.

Also, no studies of CP decontamination have been reported in the scientific literature on *Fusarium proliferatum*; there are only some mentions of this species as part of natural seed microbiota after CPT [89]. Yet *F. proliferatum* is a vital pathogen infecting crops and fruits, especially in warmer climates, and is a source of many mycotoxins [88], therefore a species of particular economic interest. Our results of both evaluation methods indicate it is probably the second most tolerant species to CPT after *Fusarium fujikuroi*: which is not surprising, as they are very similar species, both belonging to the *Fusarium fujikuroi* species complex and challenging to distinguish morphologically and genetically [90]. Therefore, it is expectable that the effect of CPT on their spores is similar. However, our results suggest that *F. fujikuroi* seems more tolerant to CPT, probably owing to the minute differences in spore structure. There are a few reports regarding the CPT of artificially infected rice seeds with *F. fujikuroi*, as this species is a primary rice pathogen responsible for rice bakanae disease. More than 90% decontamination efficacy of seeds was achieved after 120 s CPT with DBD air plasma and a significant reduction in disease development after 10 min CPT [91].

Similarly, the percentage of seedlings with disease symptoms reduced to around 8% of the control group after 10 min treatment with an air plasma jet [92]. Approximately 80% decontamination of seeds was also achieved using underwater arc discharge plasma [93]. In our study, although there were statistically significant differences already after 30 and 60 s CPT by CFU method (around 0.5 and almost 1.5-log CFU g^−1^ reduction, respectively), the difference became relevant in terms of contamination rate only after 120 s CPT, when more than 2.5-log CFU g^−1^ reduction yielded around 35% reduction in grain contamination. No further reduction was achieved after 180 s CPT. All these studies indicate that *F. fujikuroi* is probably very resistant to CPT as it is hard to complete decontamination even after very long CPT times. This could be attributed to its small-sized spores (microconidia) that can hide in the rough and wrinkled surface of the grains and thus avoid the active species generated by CP. So further studies using different devices and operating parameters should be performed on this species to find the optimal method and parameters for its complete elimination.

Based on current research, it is hypothesised that CPT destroys the fungal cell wall, making it permeable and resulting in leakage of the intracellular components [8]. Furthermore, it has also been observed that CPT causes the destruction of the DNA in fungal spores [94]. Even if the CP exposure dose was not lethal to destroy the spores or hyphae, a growth delay was usually observed [42,67]. It was also found that even if the damage was non-lethal, it could lead to physiological changes, such as the accumulation of lipid bodies due to apoptosis [78]. Ergosterol biosynthesis suppression and increased keratinase activity were also found in fungi after CPT [95].

As mentioned, gaseous plasma combines chemically reactive species, charged particles and radiation. The interaction of all these species with the surface of organic matter is exothermic. Charged particles will neutralise on the surfaces regardless of their composition or structure, and the positively charged ions will be accelerated within the sheath between bulk plasma and the object immersed in plasma. Their density in weakly ionised plasma is rather low compared to neutral chemically reactive species, so they should not play a crucial role in decontamination [45]. The density of neutral reactive species in carefully designed plasma systems is often 4–6 orders of magnitude larger than the density of charged particles [96]. In our case, the neutral radicals are oxygen atoms and OH radicals, as shown in Figure 2. We measured the density of O atoms during the treatment of buckwheat grains and found the value as significant as 2.5 × 10^21^ m^−3^. All organic materials are etched upon treatment with oxygen atoms, but the etching probability for grains is yet to be evaluated. The etching causes degradation of the spore membranes and, thus, their inactivation. Considering the inactivation curves in Figure 5 and the measured O-atom density, one may estimate the required fluence for 1 log reduction of *Aspergillus niger* of about 3 × 10^24^ m^−2^. The fluence is much larger for CP-resistant fungi. For example, Figure 5 indicates almost ten times larger fluences for *Fusarium oxysporum*. These estimates can be obtained by neglecting the interaction of OH radicals with the fungi. Unfortunately, our experimental setup was not adapted for measuring the density of these radicals.

Figure 2 shows the radiation arising from optical transitions from excited atoms or OH radicals. The radiation arises in transitions from higher excited states. However, the radiation from O and H atoms shown in Figure 2 represents only a small fraction of the total photon flux. Both oxygen and hydrogen plasma are known for extensive radiation in the vacuum ultraviolet part of the spectrum [97,98]. The radiation degrades organic materials and promotes sterilisation, but sterilisation curves for fungi using VUV photons are yet to be determined. The same applies to UV radiation at the wavelength of around 309 nm, which arises from the relaxation of excited OH radicals mainly as a transition from the first excited state to the ground level. Sterilisation using UV sources is an established technology, but most authors use mercury lamps with the most intensive line at 254 nm. Finally, the synergistic effects of ions, neutral radicals, and radiation in the broad range from near UV to VUV are likely to occur in the plasma treatment of fungi but have not been tackled so far. As mentioned earlier, the interaction between plasma species and grains is exothermic, so the thermal effects should not be neglected in any attempt to interpret the experimental results. Nevertheless, the results presented and discussed in this article give guidelines for tackling different fungi relevant to the decontamination of buckwheat grains.

From the germination tests we performed in parallel on non-autoclaved grains, our powerful low-pressure CP device decreased the germination ability of buckwheat grains already after 30 s CPT by around 13%. Only around 10% of control grains could germinate after 120 s CPT, while after 180 s CPT, germination ceased completely. We have observed that before when working with this reactor [14]. There is a challenge in selecting plasma parameters which cause adequate decontamination and are harmless for germination. However, this kind of CPT would still be suitable for postharvest treatment of buckwheat grains, e.g., for food products.

## 5. Conclusions

This study reports pioneer work on responses of a broad range of fungi colonising grains, obtained by two evaluating techniques commonly used to determine decontamination efficacy. Firstly, we showed that although grains were inoculated with the same concentration of conidia, their attachment rate was quite different, so this should always be considered in experiments comparing various fungal species which have different spores. Secondly, we showed that the method used for evaluation, could influence the reported results. Although the trends were relatively similar, we observed some minor differences that can have a biological relevance when considering contaminated grains. Finally, and most importantly, our results show that fungi with smaller spores (microconidia) could have an advantage against species with larger spores (macroconidia) when treating artificially contaminated grains with CP. Other spore structural characteristics, such as thickness and pigmentation, probably also influence the decontamination efficacy, so, further research should focus on the structural and molecular differences among species that may be essential for the outcome of the sterilisation process.

## Figures and Tables

**Table 1:** Colony forming units (CFU) in control and after different cold plasma treatment (CPT) exposure times for each fungus expressed in log CFU g^−1^ grains. Different letters (superscripts) indicate statistically significant differences between each fungus’s control and respective CPT. AA—*Alternaria alternata*; AF—*Aspergillus flavus*; AN—*Aspergillus niger*; CL—*Cladosporium cladosporioides*; EN—*Epicoccum nigrum*; FF—*Fusarium fujikuroi*; FG—*Fusarium graminearum*; FO—*Fusarium oxysporum*; FP—*Fusarium proliferatum*; FS—*Fusarium sporotrichioides*.

Fungi	Control	30 s CPT	60 s CPT	120 s CPT	180 s CPT
AA	3.57 ± 0.1 ^a^	3.21 ± 0.1 ^a^	0.77 ± 0.8 ^b^	0.0 ± 0.0 ^c^	0.0 ± 0.0 ^c^
AF	4.18 ± 0.1 ^a^	3.01 ± 0.1 ^b^	0.67 ± 0.7 ^c^	0.0 ± 0.0 ^c^	0.0 ± 0.0 ^c^
AN	4.41 ± 0.0 ^a^	1.33 ± 0.7 ^b^	1.33 ± 0.7 ^b^	0.0 ± 0.0 ^b^	0.0 ± 0.0 ^b^
CL	3.98 ± 0.0 ^a^	2.43 ± 0.1 ^b^	0.67 ± 0.7 ^c^	0.0 ± 0.0 ^c^	0.0 ± 0.0 ^c^
EN	3.91 ± 0.1 ^a^	3.62 ± 0.0 ^a^	1.33 ± 0.7 ^b^	0.0 ± 0.0 ^b^	0.0 ± 0.0 ^b^
FF	6.34 ± 0.1 ^a^	5.85 ± 0.1 ^b^	4.99 ± 0.0 ^c^	3.70 ± 0.0 ^e^	4.0 ± 0.1 ^d^
FG	3.58 ± 0.0 ^a^	0.0 ± 0.0 ^b^	0.0 ± 0.0 ^b^	0.0 ± 0.0 ^b^	0.0 ± 0.0 ^b^
FO	5.68 ± 0.0 ^a^	5.44 ± 0.0 ^b^	4.50 ± 0.0 ^c^	3.96 ± 0.0 ^d^	2.46 ± 0.1 ^e^
FP	5.94 ± 0.1 ^a^	5.70 ± 0.1 ^a^	4.78 ± 0.0 ^b^	2.80 ± 0.1 ^c^	2.93 ± 0.1 ^c^
FS	5.27 ± 0.0 ^a^	4.40 ± 0.0 ^b^	3.99 ± 0.0 ^c^	2.26 ± 0.1 ^e^	2.71 ± 0.1 ^d^

**Table 2:** Germination rate (expressed in % of germinated grains) in control and after different cold plasma treatment (CPT) exposure times. Different letters (superscripts) indicate statistically significant differences between different groups.

Treatment	Germination Rate [%]
Control	83.0 ± 4.1 ^a^
30 s CPT	72.0 ± 3.4 ^b^
60 s CPT	55.0 ± 5.2 ^c^
120 s CPT	8.0 ± 3.4 ^d^
180 s CPT	0.0 ± 0.0 ^d^

## Data Availability

All data presented in this study are available in this paper.
